# Identification of mumps virus protein and lipid composition by mass spectrometry

**DOI:** 10.1186/s12985-016-0463-0

**Published:** 2016-01-14

**Authors:** Marija Brgles, Maximilian Bonta, Maja Šantak, Maja Jagušić, Dubravko Forčić, Beata Halassy, Günter Allmaier, Martina Marchetti-Deschmann

**Affiliations:** University of Zagreb, Centre for Research and Knowledge Transfer in Biotechnology, Rockefellerova 10, HR-10000 Zagreb, Croatia; Center of Excellence for Viral Immunology and Vaccines, CERVirVac, Rijeka, Zagreb, Croatia; Vienna University of Technology, Institute of Chemical Technologies and Analytics, A-1060 Vienna, Austria

**Keywords:** Mumps virus, Proteome, Lipidome, Vero cells, Chicken embryo fibroblasts, Mass spectrometry

## Abstract

**Background:**

Mumps virus is a negative-sense, single stranded RNA virus consisting of a ribonucleocapsid core enveloped by a lipid membrane derived from host cell, which causes mumps disease preventable by vaccination. Since virus lipid envelope and glycosylation pattern are not encoded by the virus but dependent on the host cell at least to some extent, the aim of this work was to analyse L-Zagreb (L-Zg) mumps virus lipids and proteins derived from two cell types; Vero and chicken embryo fibroblasts (CEF). Jeryl Lynn 5 (JL5) mumps strain lipids were also analysed.

**Methods:**

Virus lipids were isolated by organic phase extraction and subjected to 2D-high performance thin layer chromatography followed by lipid extraction and identification by matrix-assisted laser desorption/ionization mass spectrometry (MALDI MS). Virus samples were also subjected to gel electrophoresis under denaturating conditions and protein bands were excised, *in-gel* trypsinized and identified by MS as well as tandem MS.

**Results:**

Results showed that lipids of both mumps virus strains derived from Vero cells contained complex glycolipids with up to five monosaccharide units whereas the lipid pattern of mumps virus derived from CEF was less complex. Mumps virus was found to contain expected structural proteins with exception of fusion (F) protein which was not detected but on the other hand, V protein was detected. Most interesting finding related to the mumps proteins is the detection of several forms of nucleoprotein (NP), some of which appear to be C-terminally truncated.

**Conclusions:**

Differences found in lipid and protein content of mumps virus demonstrated the importance of detailed biochemical characterization of mumps virus and the methodology described here could provide a means for a more comprehensive quality control in vaccine production.

**Electronic supplementary material:**

The online version of this article (doi:10.1186/s12985-016-0463-0) contains supplementary material, which is available to authorized users.

## Background

Mumps virus is a nonsegmented, negative stranded RNA virus of the family *Paramyxoviridae*, subfamily *Paramyxovirinae*, genus *Rubulavirus* that causes mumps disease. Mumps virions are pleomorphic particles ranging from 100 to 800 nm in size [1], consisting of a helical ribonucleocapsid core surrounded by a host cell-derived lipid envelope. Full-length genomic RNA consisting of 15384 nucleotides contains 7 tandemly linked transcription units that encode open reading frames for the nucleoprotein (NP), phosphoprotein (P), V protein, I protein, matrix (M) protein, fusion (F) protein, small hydrophobic (SH) protein, hemagglutinin-neuraminidase (HN) protein, and the large (L) protein [2]. Due to the RNA editing by insertion of G nucleotides, P gene results in three mRNA transcripts corresponding to P, V and I proteins [3]. Nucleoprotein packages RNA into a nucleocapsid that is used as a template for transcription and genome replication by RNA polymerase which is composed of L and P protein [4, 5]. V protein was found to inhibit INF-β induced antiviral state [6–8]. SH protein is considered to be a membrane protein [9] with possible function in blocking TNF-α-mediated apoptosis pathway [10]. On the virus surface are HN and F proteins that are glycosylated by the host cell machinery. HN is an attachment protein that binds receptor, and the role of F protein is to drive fusion of virus and the cell membrane resulting in virus entry [11]. Virus envelope is acquired from the lipids of the host cell during budding process and as well as protein glycosylation pattern, it is not encoded directly by the virus. For paramyxoviruses, as well as for many other viruses (retroviruses, alphaviruses, rabdoviruses, orthoviruses), it has been shown that they bud predominantly from the surface of the infected cells. However, virus budding can also occur from intracellular membranes, *e.g.* human immunodeficiency virus type-1 (HIV-1) [12, 13]. Lipid composition of several viruses has been investigated so far such as HIV [14–17], murine leukemia virus [17], influenza virus [18], hepatitis C virus [19], Singapore grouper iridovirus [20], semliki forest virus [21–23], and vesicular stomatitis virus [21, 24]. These investigations revealed in some cases resemblance of the virus membrane to plasma membrane of the host cell, but in some cases also significant differences which have been described as a consequence of virus budding from lipid rafts. So, whether virus buds from a distinct membrane region and whether this is to some extent virus directed still remains an open question. Mumps virus proteins have been investigated in 1970s and 1980s using technology available at that time. Most of the mumps proteins were tentatively detected, underwent limited characterization, and were found to be similar to other paramyxoviruses but were mostly not confirmed unambiguously [25–32].

Mass spectrometry (MS) is a powerful tool in the field of biochemistry and is emerging as a core component of fundamental discoveries also in the field of virology [33]. Combination of mass spectrometry and a relatively simple but efficient method high-performance thin layer chromatography (HPTLC) has been shown as a very convenient method for lipid analysis for not too complex lipidomes [34, 35].

Virus proteome and lipidome are expressed virus features that to some extent depend on the host cell and these must have an impact on virus characteristics such as infectivity and stability. Considering live virus vaccines, these features possibly affect vaccine performance. Therefore, methods characterizing both virus proteins and lipids could be a tool in raising the level of viral vaccines quality control. The aim of this research was to analyse lipid and protein pattern of the mumps virus derived from two cell lines (Vero and chicken embryo fibroblasts (CEF)) by means of mass spectrometry. Genome of L-Zagreb (L-Zg) mumps virus has been fully characterized [36] and here we report results on L-Zg mumps virus lipids and proteins for the first time in a truly holistic approach. Obtained data indicate that lipid profile of mumps virus depends on the host cell line and interestingly viruses derived from Vero cells contain glycolipids with up to five monosaccharide units. Mumps virus proteome was found to contain more than one nucleoprotein form whose function remains to be determined.

## Results and discussion

### Mumps virus proteins

**Fig. 1:**
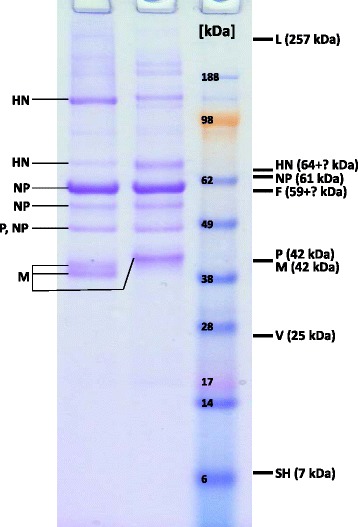
SDS-PAGE of L-Zg mumps virus grown in CEF. Left line is under non-reducing and right line under reducing conditions. Indicated on the right side are the theoretical molecular masses of mumps virus proteins. Proteins that have been identified by MS are marked on the left side. Glycoproteins are marked with „ + ?” since the glycan mass is unknown

**Fig. 2:**
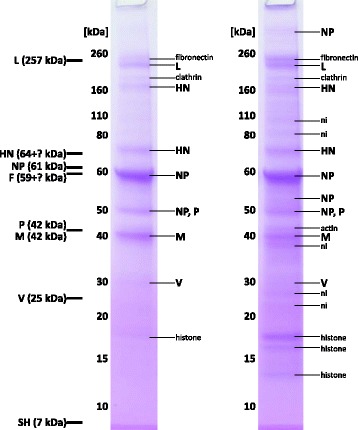
SDS-PAGE (under reducing conditions) of L-Zg mumps virus grown in Vero cells (two independent sample preparations). On the left side are theoretical mumps proteins and their calculated masses. Proteins that have been identified by MS are marked on the right side. Glycoproteins are marked with „ + ?” since the glycan mass is unknown. ni – not identified

Investigation of the most abundant mumps virus proteins was performed on L-Zg mumps virus grown on two cell substrates, CEF and Vero cells. Purified mumps virus was subjected to electrophoresis under denaturating conditions. Results show that proteins of mumps virus derived from two cell types, CEF and Vero, are similar (Figs. 1 and 2, respectively, Additional file 1: Table S1). There are more host cell proteins detected in samples derived from Vero cells but this is probably a result of limitation of the applied purification and detection techniques. Disruption of the cell caused by virus infection is variable, and is expected to have an impact on the purity of the virus. Problem of the virus isolation procedure and ability to clearly discern contaminating from interacting or constituting host cell proteins in virions has been recognized as controversial [37]. Virus purification procedure employed here consists of collecting supernatant from cells with visible cytopathic effect and removal of cell parts by low speed centrifugation followed by pelleting of viruses by ultracentrifugation. Analysis of ultracentrifugation purification procedure was found to result in 10 % recovery (n = 23) of infectivity in the pellet (determined by 50 % cell culture infective dose assay) whereas amount of the infective virus in the supernatant was below 0.01 % (manuscript in preparation). Low speed centrifugation step may not be fully effective in removal of cell proteins that are incorporated in the small parts of the cellular membrane generated during cytophatic effect caused by the viral infection of the cell, resulting in their copurification with virus particles during ultracentrifugation. Unfortunately, copurification of parts of cellular membranes with virions cannot be avoided even by density gradient purification due to the possibility of similar sedimentation coefficient. However, host cell proteins can be incorporated into virions simply by being fortuitously present at the site of budding or preferentially incorporated into virions thereby influencing viral biology and pathogenesis [38] and host cell proteins in virions have been reported for various viruses [37–44]. During our work, several virus preparations were purified and found to vary regarding amount of cell proteins. Host cell proteins that were detected are fibronectin, clathrin, actin, histones, heat shock proteins (HSP), annexins, tubulin, ribosomal protein, myosin, and all these proteins were reported to be detected in other viruses also [37–44]. Some early papers on sucrose gradient purified mumps virus particles report that mumps virions contain actin [25, 28, 29]. For respiratory syncytial virus, also a paramyxovirus, association with HSP70, HSP90 and actin among other host cell proteins has been reported [44]. Role of actin in virus morphogenesis of several viruses is described [45] as well as its role in respiratory syncytial virus maturation [46] therefore similar function could be expected in mumps.

Out of nine proteins coded by seven mumps genes, only four, HN, NP, M and P, were identified in mumps virus grown in CEF, whereas in mumps virus grown in Vero, additionally L and V proteins were detected (identification data are listed in Additional file 1: Table S1). HN protein was detected in two and NP in three bands.

Proteins not detected and identified in any of our tested samples in purified virus preparations were F, SH and I protein. I protein is not considered as a structural protein so its presence in purified virus preparations is not expected. SH protein was detected in virus infected cells and is considered to be a membrane protein [9, 10] but has not yet been detected in mumps virions. F protein is synthesized as a precursor molecule F_0_ that is processed by host cell proteases to yield active F protein composed of two disulphide-linked polypeptides [47]. Processing of the F protein has been well studied on cell lysates and F protein was detected by immunoprecipitation of the disrupted virus by anti-F antibodies [31] and in case of radioactively labelled glycoproteins in purified mumps virions [26]. However, by means of the techniques applied here we could not detect the F protein so far. Possible reasons for the lack of F protein detection could be its very low expression level, glycosylation level of the peptides (not covered by the applied MS method), low extraction efficiency from the gel matrix or peptide ion suppression, or the combination of all these factors. Interestingly, unsuccessful detection of F protein was reported for measles protein (also a member of *Paramyxoviridae*) and also multiple NP forms were confirmed [48] same as found in our work on mumps virus.

**Fig. 3:**
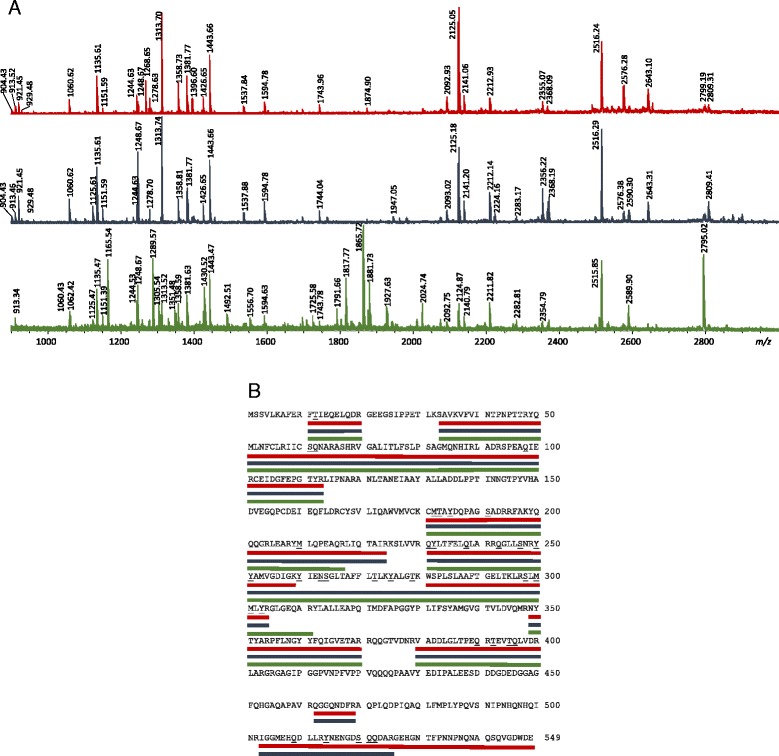
**a** Peptide mass fingerprint (based on positive ion MALDI reflectron MS) of NP forms (depicted in Fig. 1, reduced sample). Green (bottom) mass spectrum – NP apparent molecular mass 48 kDa; blue (middle) mass spectrum – NP apparent molecular mass 55 kDa; red (top) mass spectrum – NP apparent molecular mass 61 kDa. (full list of peptides with matching theoretical peptides is given in Additional file 1: Table S1). **b** Sequence coverage of NP bands from Fig. 1. Red line – NP apparent molecular mass 61 kDa; blue line – NP apparent molecular mass 55 kDa; green line – NP apparent molecular mass 48 kDa. Possibly modified amino acids are underlined

In mumps virus derived from CEF, NP has been detected in three bands and the band with the highest apparent molecular mass is most intensive and corresponds to theoretical (calculated from amino-acid sequence) NP molecular mass around 61 kDa, whereas the other two have lower molecular mass, around 55 and 48 kDa (Fig. 1). Peptide mass fingerprint (PMF) spectra of all three NP forms were analysed (Fig. 3, full peptide list in Additional file 1: Table S2) and it can be seen that spectra of NP bands with lower apparent molecular masses are missing C-terminal peptides. The same was found for mumps virus grown in Vero (Fig. 2). Presence of two NP bands in Fig. 2(a), instead of three as in Fig. 2(b) is presumably due to the limitation of the CBB detection and the present sample amount. Again, analysis of PMF was performed (Additional file 1: Figure S1, Table S3) and the C-terminal tryptic peptide was only present for the NP band of highest molecular mass, the same as with the CEF sample. Additionally, in Fig. 2(b) NP was also detected above 260 kDa marker and this is most likely an aggregated form of NP. The lowest of these protein bands, at 48 kDa, in addition to NP contained also P protein peptides and this molecular mass corresponds to the theoretical mass of P protein of 42 kDa. It should be stressed that a peptide’s occurrence in mass spectra can be influenced by various factors; trypsinization efficiency, selectivity during peptide isolation, MS inherent difficulties like ion suppression by presence of other peptides and selective desorption/ionization [49, 50]. Indeed, PMFs of these three NP bands do not contain completely identical sets of NP peptides. The lowest band contains peptides corresponding to P protein and their presence could possibly suppress some NP peptides. Nevertheless, when molecular masses of these NP proteins are calculated from sequences covered by the MS detected peptides, these data are in line with the experimentally obtained apparent molecular masses of NP bands. For the lowest NP band peptides detected in the PMF spectrum showed peptide coverage up to amino acid 400 (Fig. 3(b)). For the NP band with apparent molecular mass of 55 kDa detected peptide coverage ends with amino acid 525. Only for the NP band with apparent molecular mass of 61 kDa peptide from the C-terminus has been detected so this would indicate that NP bands with lower molecular masses are C-terminally truncated NP protein forms. Additionally, mumps virus proteins were detected also using anti-mumps serum in Western blot. The anti-mumps serum recognized three protein bands between 50 and 60 kDa and these correspond to MS identified NP forms (Additional file 1: Figure S2). Detection of only NP by the anti-mumps serum is probably a result of immunodominance of the NP as the most abundant mumps protein. There are reports on two forms (66 and 61 kDa) of mumps NP (Enders strain) detected with NP-specific monoclonal antibodies [51]. The 61 kDa form was speculated to be a product of cell proteases activity [51]. There is also one report on a truncated NP form spanning amino-acid sequence 331–498, found in cells infected with influenza virus [52]. Occurrence of this truncated NP follows kinetics of full length NP and there are no other NP forms detected with antibodies against the N-terminal part of NP protein. In addition, site at which proteolytic action should occur to result in this truncated form is not characteristic for any known endoprotease so this truncated form is reported as a likely result of transcription of one of the open reading frames [52]. Another example of truncated NP is in human influenza virus where the NP was found susceptible to cleavage by host cell caspase resulting in removal of the N-terminal peptide, and both NP forms have been found in infected cells and virions [53, 54]. The role of N-terminal caspase cleavage of influenza NP correlates with the host origin of the virus and is speculated to be a molecular determinant for host range [55]. The source and role of here detected NP forms that seem to be C-terminally truncated remains to be determined. It is known that the C-terminal part of NP in the subfamily of *Paramyxovirinae* is largely unstructured varying widely in length and sequence composition [4]. Open reading frames can result in alternate NP forms but the longest such form for mumps virus would have only 88 amino acids so this option is excluded. Other possibilities are editing of transcripts by RNA polymerase or hydrolysis by some cell protease.

M protein was detected in two very closely neighbouring gel bands under non-reducing conditions around 40 kDa corresponding nicely to the theoretical molecular mass of M protein. One band of a slightly higher apparent molecular mass was detected under reducing conditions. MS sequence analysis of M protein-derived peptides confirmed a protein modification, the acetylation of the N-terminus. M protein is one of the three most intensive proteins of mumps virus detected under experimental conditions used here, in addition to HN and NP, which is expected since it is a structural protein linking nucleocapsid to the lipid envelope.

According to literature HN protein is a disulphide bonded oligomer [2], thought to form tetramers as a mature protein [56–58] and was detected as dimer [32] and dimer and tetramer in addition to monomer form [31] in purified mumps virus samples. Here, HN protein was identified in two protein bands (around 70 and 160 kDa) that roughly correspond to theoretical monomer and dimer forms of HN taking into consideration that the molecular mass of HN calculated from amino-acid sequence is 64 kDa and that HN is glycosylated resulting in higher molecular mass. Electrophoresis under reducing conditions resulted in more intensive gel bands for the monomer in comparison to non-reducing conditions (Fig. 1) but the dimer did not disappear completely. These results indicate that in mumps virus particle a part of HN is present as a monomer and part as a disulphide bridged dimer.

In addition to P protein that was detected in protein band together with smallest NP form (in both CEF and Vero samples), L protein was also detected (in Vero samples), both at apparent molecular masses corresponding to the theoretical ones. V protein was also detected (in Vero samples) but as a faint band. V protein has not been shown as a part of mumps virion so far but this could be a result of limitation of the techniques used, it was only detected in cell lysate [30].

### Mumps virus lipids

Lipid classes present in two mumps virus strains, L-Zg and Jeryl Lynn 5 (JL5), grown in two cell lines, CEF and Vero, were analysed as well as lipids present in non-infected cells. Lipids were isolated by organic extraction, separated by two-dimensional high-performance thin layer chromatography (2D-HPTLC), visualised by primuline staining, extracted from the excised chromatographic plate material and analysed by MALDI MS. This methodology is relatively straightforward and provides wealth of data, however it is only a qualitative approach providing a global view on present lipid classes which does not detect lipids with masses below *m*/*z* 500.

**Fig. 4:**
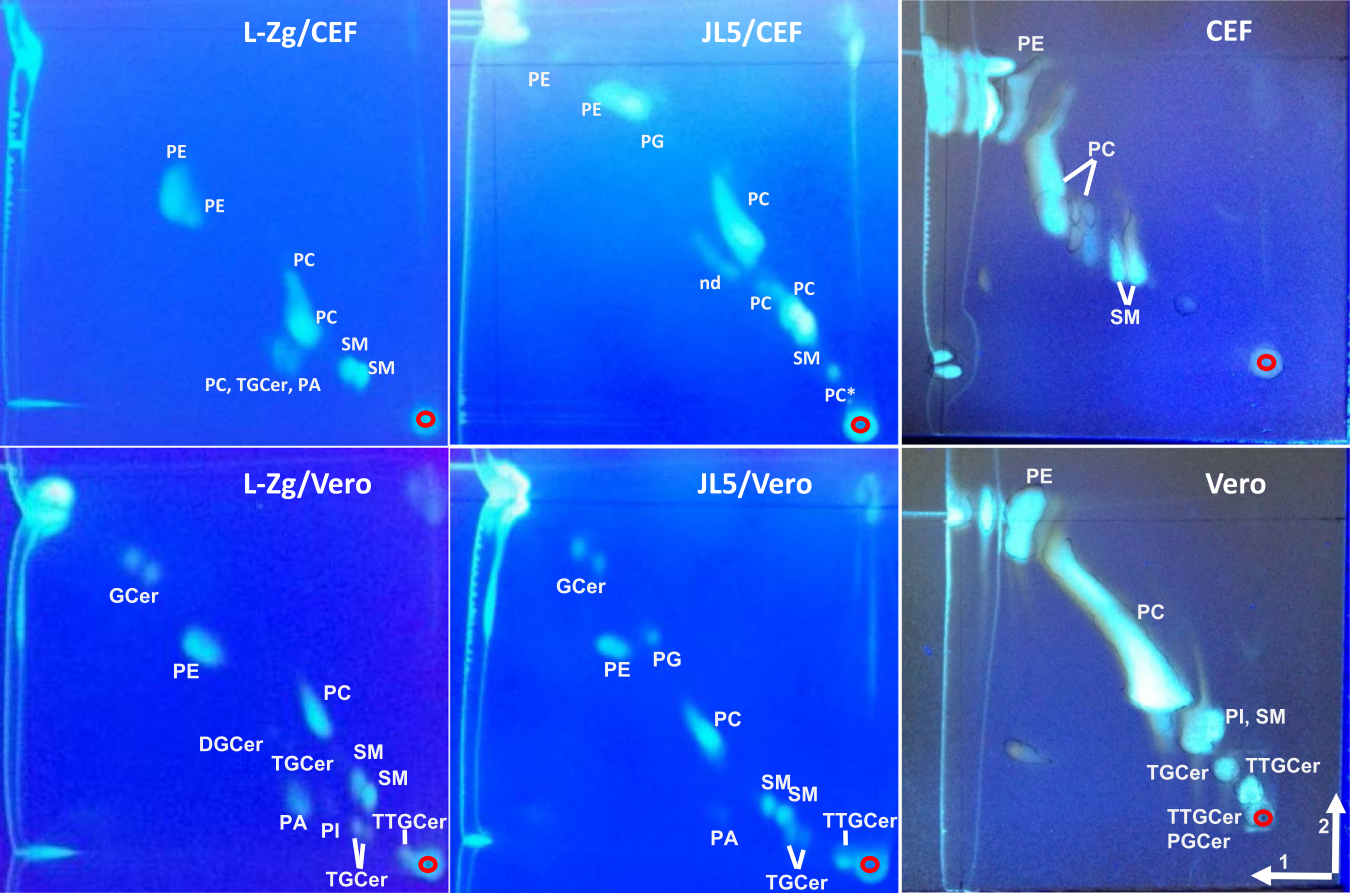
2D-HPTLC of lipids isolated from two strains of mumps virus; L-Zg and JL5, grown in Vero and CEF cells, and total cell lipids. SM - sphingomyelin, PC -phosphatidylocholine, PC* - phosphaditylcholine one chain, PGCer - pentaglycoceramide, TTGCer - tetraglycoceramid, TGCer - triglycoceramide, DGCer - diglycoceramide, Gcer - glycoceramide, PA - phosphatic acid, PE - glycerophosphoethanolamine, 2 - sphingomyelin, 3 - glycerophosphocholine, PG - phosphatidylglycerol, PI – phosphatidylinositol, nd - not determined. Development directions are denoted in the bottom right corner and point of sample application by red circles

**Fig. 5:**
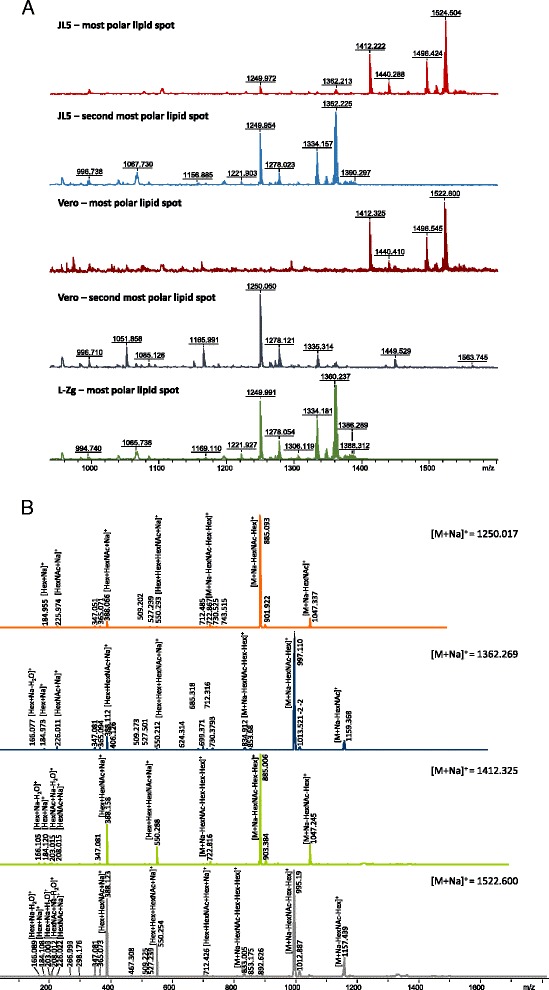
**a** Positive ion MALDI mass spectra (reflectron mode) of most polar lipids (glycolipids) extracted from L-Zg and JL5 mumps virus (grown in Vero cells) and Vero cells. **b** PSD spectra of pentaglyco and tetraglycolipids isolated from mumps virus grown on Vero cells. *m*/*z* values of parent ions are indicated on each spectrum. Hex – hexose, HexNAc- N-acetylhexose

Representative 2D-HPTLC separations of mumps virus lipids and lipids isolated from cells are given in Fig. 4. It can be seen that mumps virus derived from CEF cells has a lower number of present lipid classes compared to viruses grown in Vero cells especially regarding glycolipids. Similar results were observed for total cell lipids. The overall lipid composition of mumps virus samples is characterized by lipids also found for other viruses such as phosphatidylcholine, phosphatidylethanolamine, sphingomyeline, phosphatidic acid, glycerophosphocholine, phosphatidylinositol and glycolipids [14, 15, 17–24, 59]. Interestingly, in virus samples derived from Vero cells we detected glycolipids with up to five monosaccharide units (the most polar compounds detected on HPTLC plates). Representative mass spectra and PSD spectra of glycolipids are given in Fig. 5. The importance of glycosylation in virulence and immune interactions on the level of glycoproteins is quite well known [60–62] but the impact of glycolipids on virus properties has not been studied yet and their relevance could be equally important. Some reports on lipidomes of other viruses did not detect any glycolipids [17], some detected only glycolipids with one or two monosaccharide moieties [14, 15, 19, 20] but there are no reports on such complex virus glycolipids as detected in this study for mumps virus derived from Vero cells. The reason for this might be the methodology applied in other studies possibly omitting the separation. Or such glycolipids were actually absent since all reports discuss different cell lines possibly lacking these glycolipids. Difference in glycolipid content for different types of cells is demonstrated by our results showing that *e.g.* CEF do not contain glycolipids in contrast to Vero cells. One could presume that such complex glycolipids on the viral surface result in significant differences during *e.g.* cell infection and this we plan to investigate in our future work.

## Conclusions

Presented data on mumps virus lipids and proteins indicate the presence of host cell proteins in mumps virions as found for other viruses. Interestingly, three NP forms were detected, two of which appear to be C-terminally truncated NP proteins of yet unknown function. Lipid analysis of mumps virus showed lipid pattern to be host dependent and viruses derived from Vero cells were found to contain glycolipids with up to five monosaccharide units. Presented results demonstrate virus variability and impact of these differences on virus characteristics remains to be determined.

## Methods

### Cell lines and viruses

Vero cells (African green monkey kidney cells) were purchased from ATCC and maintained in Minimum Essential Medium with Hank’s salts (MEM-H) (AppliChem) supplemented with 10 % foetal calf serum (FCS) (Moregate) and 50 μL/mL neomycin (Gibco-Life Technologies). Chicken embryo fibroblasts (CEF) were prepared from the 11-day old embryonated chicken eggs as described [63]. L-Zg mumps virus strain was obtained from the Institute of Immunology Inc., Zagreb, Croatia. The virus strain JL5 was kindly provided by B. K. Rima (Queen's University of Belfast, United Kingdom).

Both viruses, L-Zg and JL5, were grown in Vero cells and CEFs in MEM-(H) with 2 % FCS at m.o.i. 0.0001. After 24 h the medium was replaced with medium without serum and virus was further grown until the cytopathic effect was observed. Then the medium was collected and centrifuged at 3000 × *g* to remove large-sized contaminants, and after that viruses were ultracentrifuged at 142,000 × *g* for 2 h and the pellet was resuspended in phosphate-buffered saline.

### Protein analysis

Electrophoresis under denaturating conditions was performed using 8 cm 4–12 % Bis-Tris precast gels, using MES running buffer and Novex Sharp Standard, in an XCell Sure Lock system from Invitrogen (Carlsbad) according to manufacturer’s instructions. Western blot detection was performed using guinea pig serum against mumps virus which was cultured in Vero cells and purified by ultracentrifugation. ECL Prime Western Blotting Detection Reagent was used for detection, according to the manufacturer’s instructions (GE Healthcare). Detection of protein bands was performed using acidic Coomassie Brilliant Blue R250 solution. Protein bands were excised from the gel, trypsinized and peptides isolated and purified for MS analysis as described [64]. Experiments were performed with at least two sample replicates.

### Lipid analysis

Lipid isolation was performed according to Bligh and Dyer [65]. Lipids were dried *in vacuo* and separated by 2D-HPTLC (silica plates, 5 × 5 cm, particle size 4–8 μm, Merck) using chloroform/methanol/conc. ammonia 10:5:1 (*V*/*V*/*V*) for first dimension and after air-drying by means of chloroform/methanol/acetic acid/water 15:3:2:0.6 (*V*/*V*/*V*) for second dimension. Lipids were detected using primuline solution (0.05 % solution of primuline in acetone/water 8:2 (*V*/*V*)) and 365 nm UV light. Lipid spots were scratched from plates and 250 μL of chloroform/methanol/water 10:10:0.1 (*V*/*V*/*V*) was added in order to extract the lipids and extraction procedure was repeated two times. Obtained extracts were pooled and dried *in vacuo*. Lipids were dissolved in 4 μL of 20 mg/mL 2,4,6-trihydroxyacetophenone solution in methanol and applied to a stainless steel MALDI (matrix-assisted laser desorption/ionization) -target. Ricinus oil (0.5 μL in 1 mL of methanol with addition of small amount of NaCl) was used for mass calibration of the MS instruments. Experiments were performed at least with three separate samples.

### MALDI MS analysis

Measurements were performed either on an AXIMA TOF^2^ instrument (Shimadzu - Kratos Analytical) or an UltrafleXtreme (Bruker Daltonik) in positive, reflectron ion mode. AXIMA TOF^2^ is equipped with a 20 Hz nitrogen laser (337 nm) and was operated in the positive ion mode applying an accelerating voltage of 20 keV. Analyses by means of an UltrafleXtreme (with a 2 kHz SmartBeam solid state laser (355 nm)) were performed at an acceleration voltage of 8 kV in the positive mode.

Raw data generated on AXIMA TOF^2^ instruments were converted into mzXML files and processed using mMass [66]. Data generated on UltrafleXtreme were processed using Flexanalysis (3.4.70) and Biotools (3.2_SR4). Identification searches were performed against NCBInr database, “other viruses”, with the following fixed parameters: precursor ion mass tolerance and product ion mass tolerance of ± 0.6 Da and ± 1.2 Da for AXIMA TOF^2^, ± 200 ppm and ± 200 ppm for UltrafleXtreme (respectively), two missed trypsin cleavages, carbamidomethylation of Cys and with variable modification: oxidation of Met, ammonia loss from N-terminal Cys, N-acetylation, deamidation, and phosphorylation. Data on lipids were analysed using LIPID MAPS Structure Database (LMSD) [67].

## Additional file

Additional file 1: Table S1. Identification of protein bands shown in Figs. 1 & 2. **Table S2.** List of peptides found in spectra corresponding to NP bands shown in Fig. 1 and possible matching NP and P peptides. **Table S3.** List of peptides found in spectra corresponding to NP bands shown in Fig. 2 and possible matching NP and P peptides. **Figure S1.** Peptide mass fingerprint by means of positive ion MALDI reflectron MS of NP forms (depicted in Fig. 2b, reduced sample). Green (bottom) spectrum – NP apparent molecular weight 48 kDa; blue (middle) spectrum – NP apparent molecular weight 55 kDa; red (top) spectrum – NP apparent molecular weight 61 kDa. Full peptide list is given in Table S3. **Figure S2.** Western blot of mumps virus samples; L-Zg/Vero, JL/Vero, JL/CEF (from left to right) subjected to electrophoresis under reducing denaturating conditions detected by anti-mumps serum. (PDF 612 kb)
