# Supplementary material for: Identification of mumps virus protein and lipid composition by mass spectrometry
Source: Virol J. 2016 Jan 14;13:9. doi: 10.1186/s12985-016-0463-0 (PMC4712546; doi:10.1186/s12985-016-0463-0)
Supplement: Additional file 1: Table S1. — Identification of protein bands shown in Figs. 1 & 2. Table S2. List of peptides found in spectra corresponding to NP bands shown in Fig. 1 and possible matching NP and P peptides. Table S3. List of peptides found in spectra corresponding to NP bands shown in Fig. 2 and possible matching NP and P peptides. Figure S1. Peptide mass fingerprint by means of positive ion MALDI reflectron MS of NP forms (depicted in Fig. 2b, reduced sample). Green (bottom) spectrum – NP apparent molecular weight 48 kDa; blue (middle) spectrum – NP apparent molecular weight 55 kDa; red (top) spectrum – NP apparent molecular weight 61 kDa. Full peptide list is given in Table S3. Figure S2. Western blot of mumps virus samples; L-Zg/Vero, JL/Vero, JL/CEF (from left to right) subjected to electrophoresis under reducing denaturating conditions detected by anti-mumps serum. (PDF 612 kb) [file 12985_2016_463_MOESM1_ESM.pdf]

Supplementary material

Identification of mumps virus protein and lipid composition by mass spectrometry

Marija Brgles[a,b*], Maximilian Bonta[c], Maja Šantak[a,b], Maja Jagušić[a,b], Dubravko Forčić[a,b], Beata Halassy[a,b], Günter Allmaier[c], Martina Marchetti-Deschmann[c]

[a]University of Zagreb, Centre for Research and Knowledge Transfer in Biotechnology, Rockefellerova 10, HR-10000 Zagreb, Croatia

[b]Centre of Excellence for Viral Immunology and Vaccines, CERVirVac, Croatia

[c]Vienna University of Technology, Institute of Chemical Technologies and Analytics, A-1060 Vienna, Austria

*corresponding author

Dr. Marija Brgles

Centre for Research and Knowledge Transfer in Biotechnology, University of Zagreb

Rockefellerova 10, HR-10000 Zagreb, Croatia

Tel: +385 1 6414 313; Fax: +385 1 6414 103

e-mail: mbrgles@gmail.com

Table S1. Identification of protein bands shown in Figs. 1 & 2.

Fig.	protein	NCBI sequence number[1]	Identification	PMF	sequence (PSD)
1	HN (128kD)	55775571	MS/MS		SYFPSYFSNR<br>SYFPSYFSNRR<br>EFFRPVNPYNPCSGPQQDLDQR
	HN (64 kD)	55775571	MS/MS		SYFPSYFSNR
	NP	55775564	MS/MS		FTIEQELQDRGEEGSIPPETLK<br>VFVINTPNPTTR<br>VGALITLFSLPSAGMQNHIR<br>CEIDGFEPGTYR + ammonia loss (N-term C)<br>YMLQPEAQR<br>VADDLGLTPEQR<br>TEVTQLVDR<br>IGGMEHQDLLR
	NP	55775564	MS/MS		VGALITLFSLPSAGMQNHIR<br>CEIDGFEPGTYR + ammonia loss (N-term C)
	NP	55775564	MS/MS		YMLQPEAQR
	P	55775566	MS/MS		TSTPVTEFKR<br>MINRFVEKPR<br>GGPGTAPQGQTIQEEGIDGNGALAGSKER
	M	55775568			AGSQIKIPLPKPPDSDSQR + Acetyl (N-term)<br>ILSGDPSDQQITFVNTYGFIR<br>KILSGDPSDQQITFVNTYGFIR
3a	Mixture: fibronectin, L protein	544477499<br>55775572	PMF	11.6 %<br>10.0 %	
	L protein	55775572	PMF, MS/MS	14.3 %	YQVIIPFAR<br>ILPILQAYDLR<br>LTAYPSNLFYLSR<br>NFAPLPTQFIESVPYR<br>VNLTLEQLQSLTPLPTSANLVHR
	clathrin	4758012	PMF, MS/MS	15.9 %	LLYNNVSNFGR<br>LASTLVHLGEYQAAVDGAR
	HN	55775571	MS/MS		SYFPSYFSNR<br>EFFRPVNPYNPCSGPQQDLDQR
	HN[2]	55775571	MS/MS		SYFPSYFSNR<br>SYFPSYFSNRR<br>EFFRPVNPYNPCSGPQQDLDQR
	NP	55775564	PMF, MS/MS	44.6 %	VADDLGLTPEQR<br>VFVINTPNPTTR<br>CEIDGFEPGTYR + ammonia loss (N-term C)<br>FTIEQELQDRGEEGSIPPETLK

	P	55775567<br>55775567<br>46254662	PMF, MS/MS	27.9 %	TSTPVTEFKR<br>ATIIPGVAPVLIGNPEQK<br>GGPGTAPQGQTIQEEGIDGNGALAGSKER
	NP	55775564	PMF, MS/MS	22.6 %	VFVINTPNPTTR
	M	55775568	PMF, MS/MS	55 %	GVSWSPFRK<br>IPLPKPPDSDSQR<br>KTASDKEQILFEINR<br>AGSQIKIPLPKPPDSDSQR + Acetyl (N-term)<br>ILSGDPSDQQITFVNTYGFIR<br>KILSGDPSDQQITFVNTYGFIR
	V	55775565	MS/MS		NIQYPTASHQGSK<br>ATIIPGVAPVLIGNPEQK
	histone	301792130	PMF	36.1 %	
3b	NP	55775564	PMF	22.0 %	
	fibronectin	544477499	PMF	19.6 %	
	L protein	55775572	PMF	17.0 %	
	clathrin	149053757	PMF	14.9 %	
	HN[2]	55775571	PMF	5.7 %	
	HN[2]	55775571	PMF	5.7 %	
	NP[4]	55775564	PMF	35.5 %	
	NP[4]	55775564	PMF	38.8 %	
	NP[4] + P[5]	55775564	PMF	29.5 %	
	actin	40744574	PMF	39.0 %	
	M	55775568	PMF	27.5 %	KTASDKEQILFEINR
	V	55775565	PMF[4]		
	histone	545554686	PMF	50.0 %	
	histone	470363484	PMF	60.0 %	
	histone	449282347	PMF	87.0 %	

[1]in the case of multiple gi numbers corresponding to the same peptide sequence, the one from mumps L-Zagreb is written
[2]NP peptide *m/z* 2516.25 present
[3]full list of peptides is given in Table S2 of this supplement
[4]full list of peptides is given in Table S3 of this supplement
[5]by similarity with PMF Fig. 3a

Table S2. List of peptides found in spectra corresponding to NP bands shown in Fig. 1 and possible matching NP and P peptides.

**nucleoprotein CEF**			nucleoprotein amino acid sequence	MS/MS confirmed	phosphoprotein amino acid sequence
**61 kD**	**55 kD**	**48 kD**			
904.43	904.43				
913.52	913.46	913.34	298-304		
921.45	921.45		462-469		
929.48	929.48	929.3	298-304, 1 ox 94-101		
961.52	961.52	961.39	58-65		
1061.62	1060.62	1060.43	392-400	392-400	
		1062.43			
1125.61	1125.61	1125.47	196-204		
1135.61	1135.61	1135.47	209-217	209-217	
1151.59	1151.59	1151.39	209-217, 1 ox		
		1165.54			145-154
1244.63	1244.63	1244.5	49-57		
1248.69	1248.67	1248.53	199-208		
1268.65	1268.65		503-513	503-513	
1278.63	1278.7	1278.56	296-304, 1 ox, 1 phospho		
		1289.57			135-144
		1305.54			135-144, 1 ox, 1 deamidation
		1310.45			
1313.7	1313.74	1313.52	380-391	380-391	
		1351.48			
1358.73	1358.81	1358.59	11-20, 1 phospho 37-48	37-48	
1381.77	1381.77	1381.63	231-241		
1384.70	1384.78	1384.56	90-101		
1396.63	1396.68		514-525		
1426.65	1426.65	1426.43			
		1430.52			
1443.66	1443.66	1443.47	102-113	102-113	
		1492.41	58-69, 1 phospho, 1 deamidated		373-384, 2 phospho
1537.84	1537.88	1537.65	231-242		
		1556.70			
1594.78	1594.88	1594.63			
1698.68	1698.76	1698.52	181-195		
		1725.58			275-290
1743.94	1744.02	1743.78	33-48		
	1766.82				
		1791.66			

		1816.77	298-311, 2 ox, 2 phospho			
		1865.72				275-291
1874.88	1874.88		181-195, 1 ox, 2 phospho			
		1878.74				
		1881.73	21-36, 3 phospho			275-291, 1 ox, 1 deamidated
		1927.63				
1947.84	1947.05		209-224, 1 ox			
		2024.74				
2077.01	2076.12	2075.84				
2092.83	2092.85	2092.74				
2125.05	2125.18	2124.87	243-259, 1 deamidated, 3 phospho	70-89	70-89	
2141.06	2141.20	2140.79	243-259, 1 deamidated, 1 ox, 3 phospho			
2210.99	2211.17	2210.80	281-297, 4 phospho			
	2224.16					
2282.98	2283.17	2282.86				
2355.07	2355.22	2354.79	94-113, 1 deamidated	380-400		
2368.05	2368.19	2367.87				
	2371.15	2370.82				
2498.98	2499.18	2498.91				
		2510.91	260-280, 1 deamidated, 2 phospho			
2516.24	2516.29	2515.85	380-400, 1 deamidated, 2 phospho	11-32	11-32	
2576.28	2576.38	2575.92	66-89			
	2590.30	2589.90	260-280, 3 phospho, 1 deamidated			
2643.10	2643.31	2642.87	349-370			
2654.97			526-549			
		2795.02				155-183, 2 deamidated, 1 phospho
2809.31	2809.41		503-525, 3 deamidated, 2 phospho			
	2843.24		37-57, 1 ox, 3 phospho			
	2849.44					
	2875.37					

	2891.45			
2901.31	2901.42			
	3019.53			
*3359.77**				
3825.75				
3852.12				

*italicized, grey numbers denote unresolved peaks

Table S3. List of peptides found in spectra corresponding to NP bands shown in Fig. 2 and possible matching NP and P peptides.

nucleoprotein Vero			nucleoprotein amino acid sequence	phosphoprotein amino acid sequence
**61 kD**	**55 kD**	**48 kD**		
921.56	921.56		462-469	
961.65	961.65		58-65	
		1039.77		
1060.72	1060.73	1060.77	392-400	
1087.73	1087.72			
		1095.82		
1117.75				
1135.73	1135.74	1135.76	209-217	
		1143.84		
		1181.80		
	1189.80			
1196.77	1196.78	1196.83	250-259, 1 phospho	
1220.83			392-400, 2 phospho	
1244.76	1244.79	1244.82	49-57	
1248.82	1248.85	1248.85	199-208	
1278.83	1278.84		296-304, 1 ox, 2 phospho	
1313.87	1313.88	1313.90	380-391	
1358.95	1358.96	1358.97	11-20, 1 phospho	
1370.90	1370.86	1370.96		
1381.95	1381.96	1381.98	231-241	
1384.96	1384.91	1384.96	90-101	
1426.83	1426.84	1426.87		
1443.85	1443.85	1443.88	102-113	
1500.87	1500.90	1500.90		
	1554.00			
		1573.103		
	1595.10			
	1621.09	1621.10	205-217, 1 deamidated, 1 ox	
	1625.14		298-311, 1 deamidated	
	1702.17	1702.18	205-217, 1 deamidated, 1 ox, 1 phospho	
1744.26	1744.25		33-48, 1 deamidated	
		1759.23		
		1817.22	298-311, 1 deamidated, 2 ox, 2 phospho	38-55

		1844.25		
	1925.31			
		1959.31		
	1988.32	1988.32		
	2019.28			
		2026.40		
2077.47	2076.44	2076.44		
2087.40	2087.40	2087.40		
		2110.41		
2125.47	2125.49	2125.49	70-89	
2134.49				
2168.50	2168.50	2168.50		
2182.49	2182.49	2182.50		
2212.44	2212.45	2212.50	372-391	
2267.51			102-119, 1 phospho	
	2309.57	2309.60		
2354.44	2354.50	2354.54	94-113	
2368.55	2368.58			
	2415.58	2415.60		
		2472.64		
2499.57	2499.65	2499.66		
2516.62	2516.65	2516.67	11-32	
2528.75	2528.76	2528.81	0-93, 2 deamidated	
2573,64	2573.69	2573.71		
	2576.79		66-89	
2643.67	2643.70	2643.75	349-370	
2655.52			526-549	
2794.72	2794.74			
2799.79	2798.78	2798.79	349-371	
2809.75	2809.78		503-525, 3 deamidated, 2 phospho	
		2826.84	37-57, 2 deamidated, 3 phospho	154-181
	2856.85	2855.80		
	2901.99			
		3102.88		
3802.44				
3850.45			470-502	
3859.50				
3907.50				

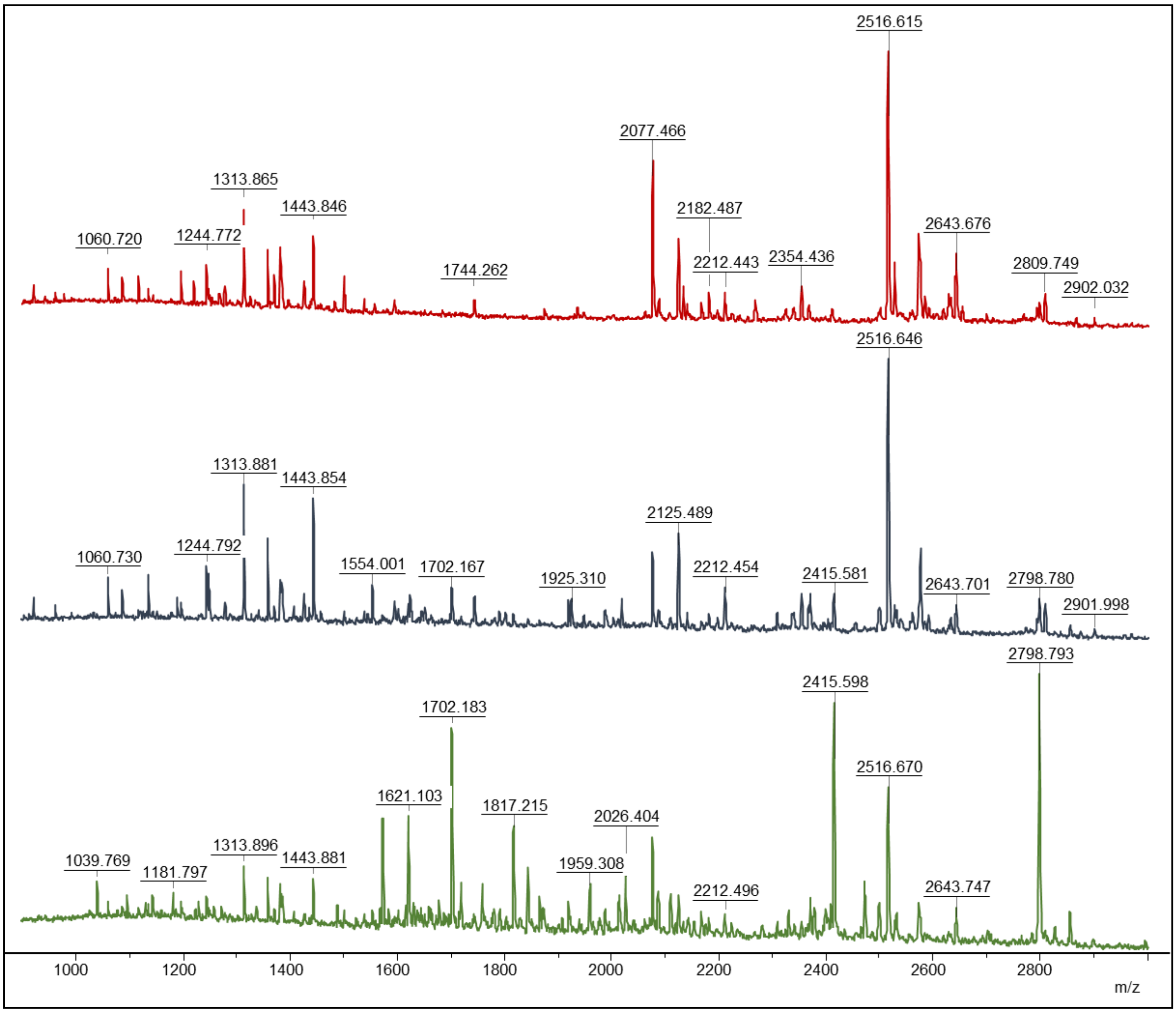

Figure S1. Peptide mass fingerprint by means of positive ion MALDI reflectron MS of NP forms (depicted in Fig. 2b, reduced sample). Green (bottom) spectrum – NP apparent molecular weight 48 kDa; blue (middle) spectrum – NP apparent molecular weight 55 kDa; red (top) spectrum – NP apparent molecular weight 61 kDa. Full peptide list is given in Table S3.

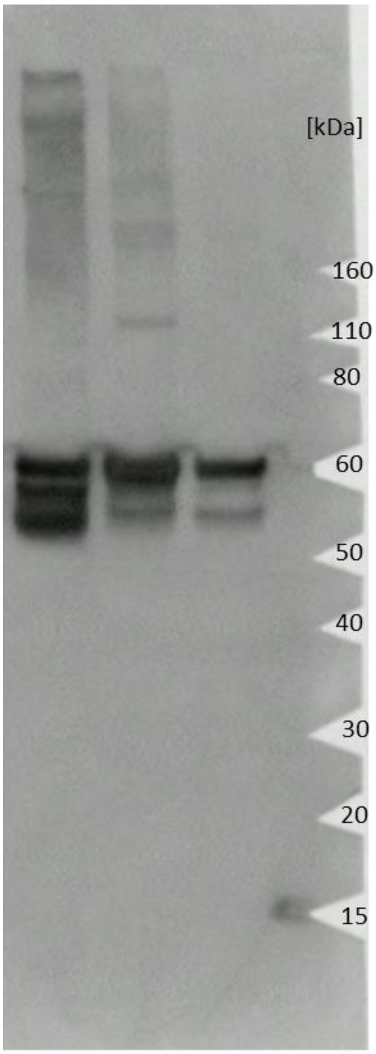

Figure S2. Western blot of mumps virus samples; L-Zg/Vero, JL/Vero, JL/CEF (from left to right) subjected to electrophoresis under reducing denaturating conditions detected by anti-mumps serum.
